# Advances in hyperbaric oxygen to promote immunotherapy through modulation of the tumor microenvironment

**DOI:** 10.3389/fonc.2023.1200619

**Published:** 2023-09-15

**Authors:** Pei Wang, Xiao-Yan Wang, Chang-Feng Man, Dan-Dan Gong, Yu Fan

**Affiliations:** ^1^ Cancer Institute, The Affiliated People’s Hospital of Jiangsu University, Zhenjiang, Jiangsu, China; ^2^ Department of Gastroenterology, The Affiliated Suqian First People’s Hospital of Xuzhou Medical University, Suqian, Jiangsu, China

**Keywords:** immunotherapy, tumor microenvironment, hyperbaric oxygen, hypoxia-inducing factors 1α, reactive oxygen species

## Abstract

Hyperbaric oxygen therapy is a relatively safe treatment method that has been used for a long time in the clinic. It has been proven that it can enhance the sensitivity of radiotherapy and photodynamic therapy for cancer. However, there are few studies on hyperbaric oxygen and immunotherapy. In this article, we summarize that hyperbaric oxygen therapy regulates the tumor microenvironment through various pathways such as improving tumor hypoxia, targeting hypoxia-inducing factors, and generating reactive oxygen species. The change in the tumor microenvironment ultimately affects the curative effect of immunotherapy. Therefore, hyperbaric oxygen can influence immunotherapy by regulating the tumor microenvironment, providing a direction for the future development of immunotherapy.

## Introduction

**Figure 1:**
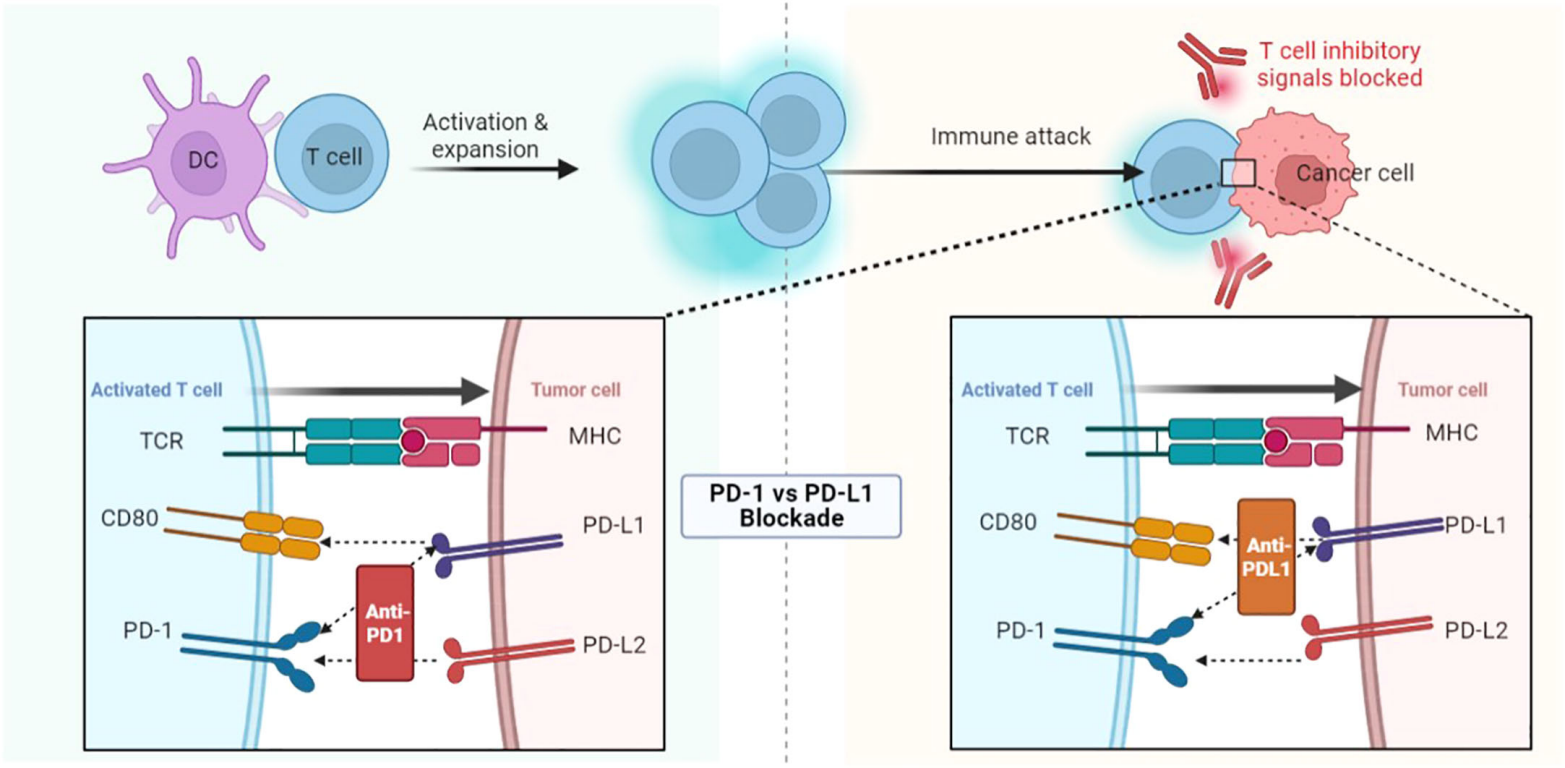
PD-1/PD-L1 antibodies target two endpoints of the same immune pathway and therefore have very different mechanisms of action and clinical efficacy. DC, Dendritic cells; TCR, T cell receptor; CD, Cluster of differentiation; PD, Programmed cell death protein; PD-L, Programmed cell death ligand; MHC, Major histocompatibility complex.

The high morbidity and mortality rate of cancer seriously affect people’s health. The treatment of tumors mainly includes surgical resection, radiotherapy, chemotherapy, targeted therapy, immune checkpoint inhibition, and so on (1). Immunotherapy is one of the successful methods. Its mechanism is to block the immune checkpoint expressed by tumor cells and enhance the killing effect of T cells (2). Immune checkpoint blockers (ICBs) mainly act on immunosuppressive targets, such as cytotoxic T lymphocyte-associated antigen 4 (CTLA-4) and programmed cell death protein 1 (PD-1), or block immune checkpoint-related ligands, such as programmed cell death ligand 1 (PD-L1). Therefore, CTLA-4 antibody and PD-1/PD-L1 antibody are the main immune checkpoint inhibitors in clinical applications (3, 4). Although PD-1/PD-L1 antibodies target two endpoints of the same immune pathway, they are quite different in mechanism of action, clinical efficacy, and drug resistance (5) (**Figure 1**). In the process of clinical application, immunotherapy has experienced unpredictable primary and acquired drug resistance (6), which has affected its promotion and sustainable application (2, 7). Immunotherapy has brought survival benefits to countless cancer patients since its advent. Therefore, overcoming drug resistance to immunotherapy is particularly important in its long-term development. At present, it has been found that the tumor microenvironment has a certain influence on immunotherapy (8, 9).

Hyperbaric oxygen (HBO) therapy, as a clinical treatment with a certain history, has been widely used in hypoxia and wound healing (10). In recent years, studies have shown that HBO can improve the curative effect of radiotherapy and photodynamic therapy for tumors (11). Whether HBO can promote T cells to enter the tumor core, improve tumor-killing activity and promote immunotherapy is still a problem worthy of study (12). This article will discuss the relationship between HBO and immunotherapy from the tumor microenvironment level, and further clarify the influence of hyperbaric oxygen on immunotherapy.

## Tumor microenvironment: (hypoxia, blood vessel, extracellular matrix, hypoxia-inducible factor 1α)

Tumor microenvironment refers to the local biological environment in which solid tumors are located, including cancer cells and their nearby stromal cells (13). In the early stage of tumors, passive diffusion is the main way for cancer cells to transport nutrients. As tumor size increases, insufficient oxygen supply and metabolic waste accumulation will cause hypoxia and acidosis in the tumor microenvironment. The hypoxic tumor microenvironment induces immature neovascularization, which leads to vascular leakage (14). Extracellular matrix (ECM), as an important part of the tumor microenvironment (15), not only provides a physical scaffold for cancer cells but also plays a key role in diffusion and drug resistance.

### Hypoxia

Hypoxia can activate hypoxia-inducible factor 1α (HIF1α) (16), which upregulates PD-L1 expression on dendritic cells and cancer cells, leading to immunosuppression (17, 18). Hypoxia also can inhibit the activity of T cells and the antigen-presenting ability of dendritic cells (19, 20). Hypoxia can induce invasive matrix molecules and increase the invasive potential of cancer cells (21). It can also up-regulate the expression of drug-resistant molecules, induce cell cycle arrest, and lead to the insensitivity of cancer cells to radiotherapy and chemotherapy (22).

**Figure 2:**
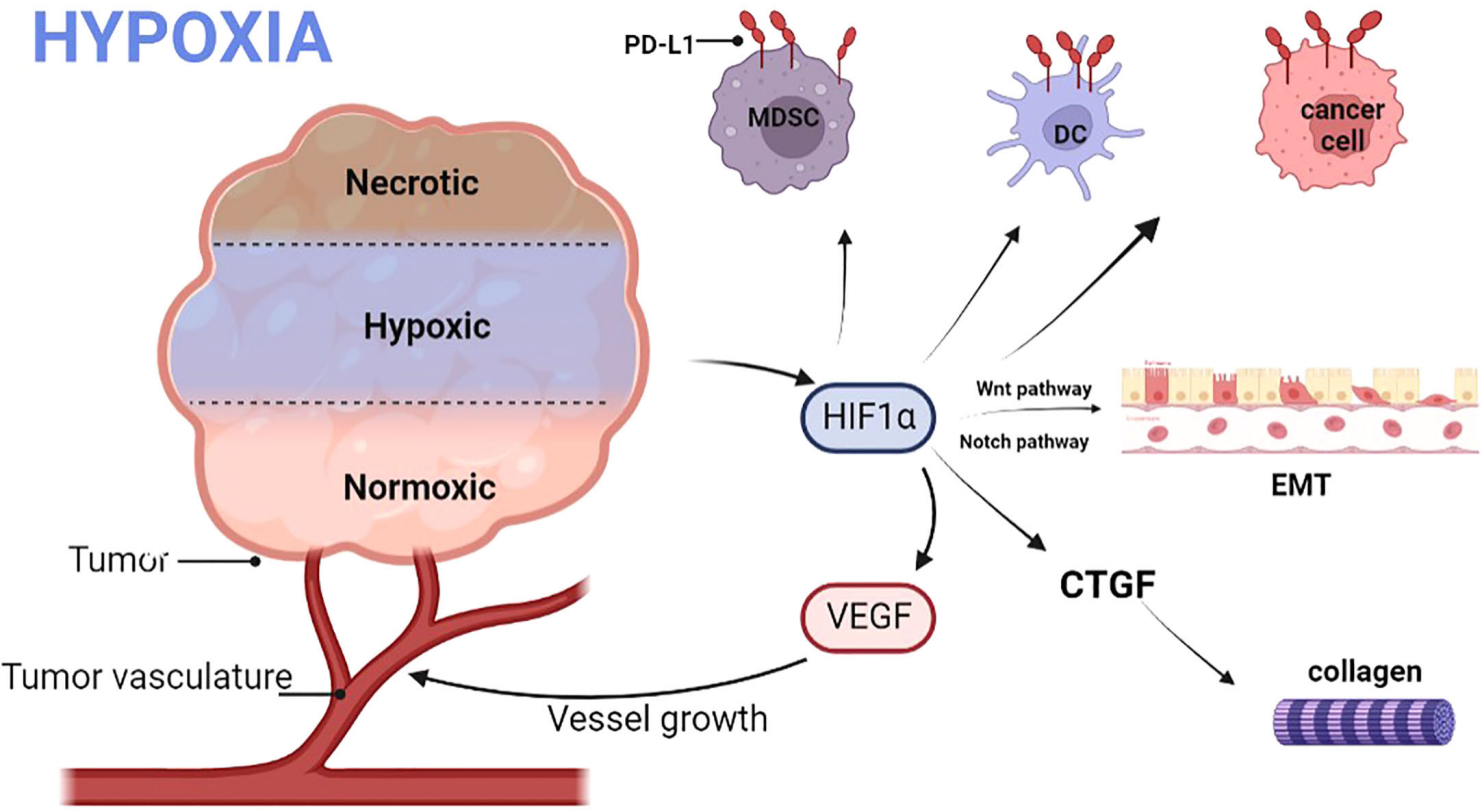
Hypoxia promotes the release of HIF, leading to an increase in VEGF, which in turn promotes the growth of tumor vessels. HIF also promotes the expression of PD-L1 on MDSC, DC, and tumor cells; promotes EMT via the Wnt and Notch pathways. Finally, HIF also promotes the production of collagen fibers. DC, Dendritic cells; PD-L, Programmed cell death ligand; MDSC, Myeloid-derived suppressor cells; HIF1α, Hypoxia-inducible factor 1α; EMT, Epithelial-mesenchymal transition; VEGF, Vascular endothelial growth factor; CTGF, Connective tissue growth factor.

Myeloid-derived suppressor cells (MDSC) are the largest group of suppressor cells in the tumor microenvironment and are considered the main obstacle to immunotherapy (23). Hypoxia can recruit immature myeloid cells and transform them into MDSC. MDSC can also be recruited by secreting chemokines (24). Hypoxia can also directly combine with PD-L1 to selectively up-regulate MDSC (18). The activation of MDSC can lead to immunosuppression (**Figure 2**).

### Blood vessel

Hypoxia can also induce vascular endothelial growth factor (VEGF) and platelet-derived growth factor (PDGF) to destroy the stability of blood vessel walls (25, 26) and induce immature neovascularization. The local high permeability of blood vessels can cause plasma to leak from blood vessels into tumor stroma, which leads to an increase in extravascular hydrostatic pressure (27) and hinders drug transportation. Most anti-cancer drugs exert selective toxicity on cells, so cells that proliferate slowly are usually drug-resistant (28). As the distance from tumor vessels increases, the proliferation of tumor cells decreases gradually, and the concentration of exposed drugs decreases, which eventually leads to drug resistance (29).

### Extracellular matrix

ECM is composed of collagen, fibronectin, and elastin, which is an important part of the tumor microenvironment. Hypoxia up-regulates HIF1α, induces connective tissue growth factor (CTGF), and regulates collagen deposition (22). Collagen deposition forms a denser ECM, which promotes the directional migration of cancer cells. ECM derived from anoxic fibroblasts was found to be 3 times stiffer than ECM derived from non-anoxic fibroblasts (30). Therefore, the dense ECM in the tumor microenvironment affects the curative effect of small molecule drugs, let alone the infiltration of Cytotoxic T lymphocyte (CTL) and PD-1 antibody (31).

### Hypoxia-inducible factor 1α

HIF1α is the core of hypoxia response (32), and it is also an important regulatory factor for cells to adapt to hypoxia (33–35). Under physiological conditions, HIF1α was easily degraded (36). When the oxygen partial pressure in the body decreases, HIF1α will accumulate (32). HIF1α is pleiotropic, including metabolic adaptation, neovascularization, and metastasis (33, 34).

Epithelial-mesenchymal transition (EMT) is a biological process in which epithelial cells are transformed into mesenchymal phenotypic cells through specific processes (37). HIF1α is the key transcription factor of EMT. Recent studies have shown that HIF1α can induce EMT, which leads to metastasis and poor prognosis of hepatocellular carcinoma (HCC) (38). Long non-coding RNA (lncRNA) can inhibit T cell immune function by affecting regulatory T cell (Treg) and PD-1/PD-L1 immune checkpoints (39). Under hypoxia, HIF1α can target lncRNA to influence immunotherapy. HIF1α activates the expression of PD-L1 by directly binding to the hypoxia response element in the proximal promoter of PD-L1. HIF1α induces VEGF and inhibits dendritic cell maturation (40, 41). VEGF down-regulates T cell function by enhancing PD-L1 expression in dendritic cells (42–44). Therefore, HIF1α may be the key factor of drug resistance in immunotherapy.

In a word, the tumor microenvironment is not only a silent bystander but an active promoter in the process of cancer occurrence (45). Studies have shown that the immune tolerance of tumors can be attributed to the tumor microenvironment of immunosuppression (46). Therefore, targeting the tumor microenvironment can enhance the effect of tumor immunotherapy to a certain extent.

## Hyperbaric oxygen

Hyperbaric oxygen therapy is based on nearly 100% pure oxygen (at least 95% oxygen) and increased barometric pressure (47). When the patient inhales 100% oxygen, the extra pressure will increase the dissolved oxygen in plasma and increase the oxygen tissue transport independent of hemoglobin (48, 49). In addition, increased barometric pressure produced by HBO therapy may exert anti-tumor biological activity through gene expression (50). This is an incomparable advantage of HBO over other oxygen delivery methods (31). HBO is often used as the main means to treat carbon monoxide poisoning, decompression sickness (51, 52), and other ischemic and hypoxic diseases. Malignant tumors were once a contraindication of HBO. More and more evidence proves that HBO has a neutral effect on malignant tumors (48, 53). Studies have shown that HBO can reduce drug resistance to chemotherapy and radiotherapy (54). In conclusion, there is no research to prove that HBO promotes cancer recurrence and metastasis so far (48, 55). In some tumor models, HBO can inhibit the proliferation of cancer cells and stimulate the apoptosis of cancer cells (49). Therefore, the role of HBO in malignant tumors needs further study.

## Hyperbaric oxygen affects the immune system

HBO therapy has broad-based effects on the immune system in normal individuals and human disease. By observing the antibody reaction of sheep erythrocytes, it was found that HBO had an immunosuppressive effect on normal mice and autoimmune mice. HBO can lead to lymphocyte death through direct oxygen cytotoxicity or endogenous steroid hormones induced by oxidative stress (56). In autoimmune diseases, HBO can selectively eliminate abnormal lymphocyte subsets, showing potential therapeutic effects (57). Shao-Yuan Chen found that HBO can reduce the deposition of immune complexes in the kidney of lupus nephropathy mice and improve the survival rate (58). After HBO exposure, the production of pro-inflammatory cytokines and the level of steady-state RNA in blood-derived monocytes were inhibited (59).

In addition, HBO can also affect immune response by regulating gene expression. Ye Chen analyzed gene expression after exposure to different levels of partial oxygen pressure and found that both independent and overlapping genes were sensitive to increased pressure and/or oxygen (60). After genome-wide microarray analysis of human microvascular endothelial cells, Godman found that up to 8,100 genes were up-regulated or down-regulated within 24 hours after exposure to HBO. The up-regulated genes are mainly growth and repair hormones and anti-inflammatory genes, while the down-regulated genes are mainly pro-inflammatory and apoptotic genes (61). Based on much literature, Paul G. Harch concluded that hyperoxia and/or atmospheric pressure have a wide range of promoting and inhibiting effects on gene expression (50). HBO activates the expression of genes that protect and promote the growth of endothelial cells and enhances the function of endothelial cells. HBO regulates the up-regulation of anti-inflammatory genes and down-regulation of pro-inflammatory genes, thus reducing inflammatory response (61). Therefore, the combination of HBO and immunotherapy may up-regulate immune genes. Finally, gene therapy plays an anti-tumor role.

## Hyperbaric oxygen regulates the tumor microenvironment (hypoxia, blood vessels, ECM)

Normobaric hyperoxia, meaning hyperoxia from breathing an increased FiO2 of oxygen at ambient atmospheric pressure. Scholars have found that normobaric hyperoxia can induce apoptosis by regulating the tumor microenvironment. Normobaric hyperoxia can enhance the anti-tumor activity of T cells and natural killer cells (NK), leading to the death of tumor cells (62). Normobaric hyperoxia provides a feasible direction for improving the immunotherapy of cancer. Both HBO and normobaric hyperoxia use oxygen to improve tumor hypoxia. Therefore, the effect of HBO on the tumor microenvironment is worth exploring.

In the mouse HCC tumor model, HBO uses oxygen to oxygenate the tumor, relieve tissue hypoxia and improve the anti-tumor effect of Doxil (22). In the pancreatic cancer model, HIF1α expression decreased after HBO (63–65). Pan Wang found that HBO enhanced the sensitivity of chemotherapy drugs by inhibiting the expression of HIF1α (66). HBO promotes immunotherapy by relieving tissue hypoxia and down-regulating PD-L1 (67).

HBO can promote angiogenesis in patients with traumatic brain injury (68–70). Katarzyna Stępień believes that HBO can be used as an adjuvant in chemotherapy to promote the development of new blood vessels and the transportation of drug molecules (49). In a mouse model inoculated with human epithelial ovarian cancer cells subcutaneously, T Alagoz found that HBO increased the efficacy of cisplatin by inducing angiogenesis (71). Cluster of differentiation (CD) 31, as a mitogenic factor in wound healing, is highly expressed in endothelial cells and related to tumor angiogenesis. Shao-Yuan Chen found that CD31 expression increased significantly 14 and 28 days after HBO treatment. HBO improved tumor angiogenesis but did not increase tumor growth (54). However, in breast cancer (72–74) and glioma models, the diameter and density of tumor peripheral blood vessels decreased significantly after HBO treatment (75). The effect of HBO on tumor vessels may depend on the tumor model, animal species, or other factors. The role of HBO in angiogenesis remains controversial.

**Figure 3:**
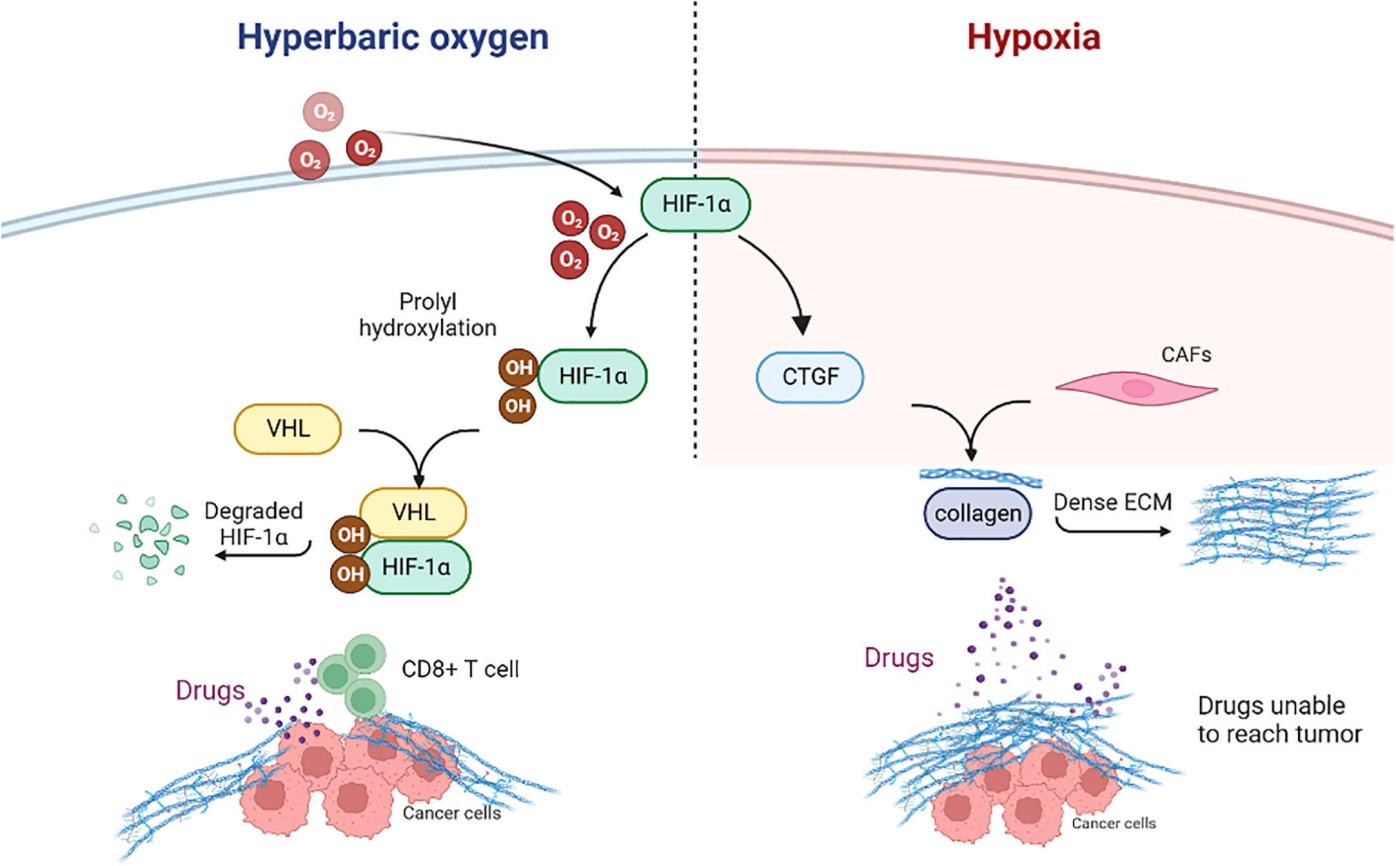
Hyperbaric oxygen can decompose dense ECM through various mechanisms. HIF1α, Hypoxia-inducible factor 1α; CTGF, Connective tissue growth factor; CAFs, Cancer-associated fibroblasts; ECM, Extracellular matrix; VHL, Von Hippel Lindau; CD, Cluster of differentiation.

Cancer-associated fibroblasts (CAFs) can produce dense ECM, which confines T cells to the matrix and inhibits the anti-tumor immunity of T cells (76). In the mouse pancreatic cancer tumor model, HBO significantly inhibited CAFs (63). After HBO treatment, the transcription and expression of CTGF and type I collagen decreased significantly, and dense ECM was decomposed. HBO can directly consume collagen fibers and fibronectin in ECM, promoting drug transport (63). In a word, HBO consumes the dense ECM around tumor cells through various mechanisms, increases the infiltration of PD-1 antibodies and T cells into tumor parenchyma (31), and promotes the immunotherapy of cancer (**Figure 3**).

HBO can reduce the number of Treg cells in tumor tissue and alleviate the immunosuppressive microenvironment (31). To sum up, HBO can target the tumor microenvironment to promote cancer immunotherapy.

## Hyperbaric oxygen targets HIF1α

In the chronic lymphocytic leukemia (CLL) mouse model, decreasing the expression of HIF1α can increase the survival rate of the CLL mouse model. HIF1α inhibitors can exert toxicity on CLL cells (33). HIF1α inhibitor has a strong anti-tumor function, and combined with ibrutinib can induce cytotoxicity (34). Therefore, targeting HIF1α is a promising therapeutic strategy.

HIF1α mediates the immune escape of various hypoxic solid tumors. Qinghua Wu et al. found that HIF1α inhibitors can reduce the expression of PD-L1 (77). Xing-Chen Ding proved that targeting HIF1α can improve the therapeutic effect of anti-PD-1/PD-L1 in glioma (78). Therefore, blocking PD-L1 and inhibiting HIF1α is a promising combination therapy (79, 80). Inhibition of HIF1α can release the anti-tumor activity of NK cells (81). Yen-An Tang found that inhibition of HIF1α can reverse chemotherapy resistance caused by tumor microenvironment (82). In a word, the HIF1α pathway plays an important role in the treatment of cancer.

HBO can inhibit HIF1α in tumors. HBO inhibits the Warburg effect, hyperproliferation, and EMT of non-small cell lung cancer cells by down-regulating HIF1α (83). In the glioma model, HBO inhibited HIF1α and improved prognosis (66). HBO can regulate the HIF1α/CTGF/type I collagen pathway (22) and improve dense ECM.

HBO can not only reduce the expression of PD-L1 (67) but also down-regulate HIF1α. Therefore, it has a positive role in promoting immunotherapy.

## Hyperbaric oxygen produces ROS

Reactive oxygen species (ROS) is an oxygen-containing molecule that protects and harms cancer cells. An appropriate amount of ROS can regulate biological function and intracellular homeostasis, while an excessive amount of ROS can induce cell death through various mechanisms (84). ROS can act as a signaling molecule and regulate EMT in many ways (85). Many studies have shown that ROS has dual effects on cancer. Therefore, we need to dialectically view the role of ROS in cancer treatment (86).

HBO can produce excessive ROS (87). In the HBO environment, photodynamic therapy can generate a large amount of ROS in hypoxic tumors. At the same time, the fluorescence intensity of HBO-treated cells was significantly higher than that of normal oxygen-treated cells, suggesting the generation of ROS (88).

ROS can be involved in the initiation and metastasis of cancer (85). ROS can also stabilize HIF1a, and cause cancer metastasis and drug resistance (89). In a glioma mouse model, HBO can induce ROS in the thymus, inhibit T cell maturation, leading to immunosuppression, and finally promote the growth of malignant glioma cells (90). There are two types of macrophages, Macrophages1 (M1) is involved in tumor killing, and Macrophages2 (M2) is involved in tumor growth and metastasis (86, 91). In lung cancer and breast cancer models, ROS is necessary for the tumor to acquire the M2 phenotype (92). ROS can promote macrophage recruitment and M2 polarization. It can inhibit T cells and NK cells, and help cancer cells escape immune surveillance and immune defense (86). Other studies have shown that ROS may reduce the effectiveness of PD-1 antibodies (93). Therefore, ROS has a certain inhibitory effect on tumor immunotherapy.

ROS can also act as an intracellular signal in the apoptosis pathway (94). Researchers found that high doses of ROS are a promising cancer treatment strategy. Adriamycin can induce apoptosis by inducing ROS in cells, and HBO can enhance its cytotoxicity. Chunle Zhao found that a large amount of ROS has a killing effect on cancer cells (84). High ROS, as a strong oxide, can induce oxidative stress and activate programmed cell death (95). For example, excessive ROS can inhibit Epidermal Growth Factor Receptor (EGFR)-mediated Phosphatidylinositol 3-kinase (PI3K)/Akt signaling pathway and block the proliferation of androgen-independent prostate cancer cells (96). ROS can also block the PI3K/AKT/nuclear factor kappa-B (NF-κB) pathway and inhibit the proliferation of non-small cell lung cancer A549 cells (86). ROS can activate p53, which leads to the arrest of the tumor cell cycle (97). ROS can enhance the antigen-presenting ability of dendritic cells, thus triggering the differentiation of monocyte precursors or hematopoietic cells and inducing their maturation (93). In addition, ROS can also reshape or degrade ECM, and serve as a target for anti-tumor therapy. The relationship between ROS production and PD-L1 expression is not clear, but ROS level affects PD-L1 expression in cancer cells (93). It has been proven that ROS combined with PDL-1 blocking can promote the presentation of tumor antigens to primitive T cells and enhance adaptive anti-tumor immunity (46). Tumor-reactive CTL was isolated from mice treated with anti-PD-L1, and it was found that CTL carried high levels of ROS, which could enhance the activity of PD-1 blockers (93). ROS can also promote intratumoral invasion of CTL and sensitize the tumor to PDL-1-blocking therapy (46). Therefore, ROS can promote immunotherapy to some extent.

The role of ROS in cancer is a double-edged sword. A certain degree of ROS can promote the occurrence and development of cancer, but excessive ROS can induce apoptosis of cancer cells through various mechanisms (84–86, 98). Therefore, HBO can have positive or negative effects on immunotherapy by producing ROS.

## The impact of the tumor microenvironment on immunotherapy

Hypoxia and HIF1α can induce immunosuppressive cells contributing to immune tolerance and forming an inhibitory immune microenvironment. Abnormal tumor vascularization can impair blood flow, aggravate hypoxia, and limit the delivery of nutrients and drugs (99). Dense ECM prevents drug penetration into the tumor core, which leads to drug resistance. Therefore, targeting hypoxia and promoting the normalization of tumor blood vessels are helpful to the efficacy of immunotherapy. HBO can regulate the tumor microenvironment and improve cancer immunotherapy by targeting HIF1α, relieving tissue hypoxia, and consuming ECM.

## Hyperbaric oxygen and other immunotherapy

Immunotherapy mainly includes ICBs, molecular targeted therapy, adoptive immune cell therapy, cytokine therapy, and tumor vaccine. Antibody therapy is one of the immune therapies. Kun Li et al. found that after teniposide chemotherapy, HBO promoted the recruitment of activated CTL, and the tumor microenvironment changed from a non-inflammatory state to an inflammatory state. HBO combined with teniposide chemotherapy increased the sensitivity of the tumor to PD-1 antibody and improved the therapeutic effect of PD-1 antibody in various tumor models (100). Ustekinumab, as an immunosuppressant, blocks the synthesis of Interleukin (IL)-12 and IL-23 and inhibits the activity of T cells. Lauren E Provini reported for the first time a case of HBO combined with ustekinumab in the treatment of severe suppurative sweat gland inflammation (101). Antivenom is a drug containing specific antibodies. The effect of HBO combined with antivenom was better than that of antivenom alone (102). Rituximab is a monoclonal antibody that targets CD20 cells. A Chinese woman with a severe vasculitis ulcer was treated with rituximab, methotrexate, and HBO, and the ulcer was improved (103). In addition, HBO can increase the curative effect of adalimumab in hidradenitis suppurativa (104). We found that HBO can increase the efficacy of antibody therapy in diseases. Therefore, these applications in other disease states are templates for possible combinations of HBO and cancer antibody therapy.

## Practical application of hyperbaric oxygen

The dose of HBO is composed of two independent components, namely hyperoxia and increased barometric pressure. HBO plays an immunomodulatory role depending on oxygen and pressure (105). T Alagoz exposed the mouse tumor model to three 30-minute HBO (100% oxygen pressurized to 2.4 atmospheres) exposures and two 10-minute air interruptions per day. After 5 days of HBO treatment, cisplatin chemotherapy was performed. Finally, T Alagoz’s team found that HBO promotes the vascular supply of tumors and helps the delivery of chemotherapy drugs (71). Ingrid Moen divided mice into three groups, one group received intermittent HB0 treatment for three days (1^st^, 4^th^, and 7^th^ days), one group received continuous HBO treatment for seven days, and one group served as a control group. HBO treatment was performed by pressurizing 100% oxygen to 2.5 bar for 90 minutes. The final results showed that only after intermittent HBO treatment, the blood vessel density decreased. At the same time, hyperoxia leads to down-regulation of the mitogen-activated protein kinase (MAPK) pathway and inhibits tumor growth (74). Metastatic mouse osteosarcoma cells were treated with HBO (100% oxygen pressurized to 2.5 atmospheres, 5 times a week for 5 weeks) and carboplatin. Yasuomi Kawasoe found that HBO enhanced the chemotherapy effect of carboplatin and significantly inhibited osteosarcoma growth and lung metastasis (106). The mouse H22 subcutaneous tumor model was treated with HBO (pure oxygen pressurized to 2.5 atmospheres) for 1.5 hours, and then the PD-1 antibody was injected intravenously. Xin Liu found that HBO enhanced the immune response of PD-1 antibody and the infiltration of T cells into tumor parenchyma (31). After Xian Wu combined the nano-drug Doxil with HBO (more than 97% oxygen pressurized to 2.5 atmospheres absolute), it was found that collagen deposition decreased and tumor hypoxia eased. Combined therapy synergistically inhibited tumor growth, and the inhibition rate reached 91%. Therefore, the combination of HBO and other nano-drugs may become a safe way to treat tumors (22). Pan Wang used BALB/c-nu mice to inoculate glioblastoma cells into the brains of mice. Mice were injected with temozolomide and exposed to HBO (2.5 atmospheres of pure oxygen) for 90 minutes. The results showed that HBO treatment alone might promote tumor growth. The tumor volume of mice in HBO combined with the temozolomide group decreased and the survival time was prolonged. HBO combined with temozolomide can inhibit HIF1α and HIF2α expression and promote chemical sensitization (66). Xiaoxian Wang used HBO (pure oxygen pressurized to 2.5 atmospheres absolute) in combination with Abraxane, and gemcitabine. HBO inhibits CAFs, normalizes tumor vessels, and enhances the anti-tumor activity of drugs (64). Shao-Yuan Chen exposed metastatic cells to HBO (98% oxygen, 2.5 atmospheres absolute). As a result, HBO improved tumor vascular hypoxia and targeted tumor apoptosis-related genes (54). After HBO (> 97% oxygen, pressure 2bar) treatment, tumor vessel density decreased and tumor cell apoptosis increased (75). Yong-Gang Wang established a mouse glioma model. After HBO (100% oxygen, 2.5 atmospheres) treatment, the ROS level was evaluated by flow cytometry. They found that HBO reduced ROS levels in brain cells and raised ROS levels in the thymus. Finally, it inhibits T-cell maturation and promotes the growth of malignant tumors (90). Chunxia Chen found that ROS and lipid ROS levels in HT22 cells and PC12 cells decreased after HBO (pure oxygen, 0.25 MPa) treatment, thus protecting cells from oxygen-glucosedeprivation (107). However, Qin Hu et al. found that delaying HBO (2.5 atmospheres absolute) significantly increased ROS level, which may improve the long-term rehabilitation of stroke patients through the ROS/HIF-1 α/β-catenin pathway (108).

In short, in the process of practical application of HBO, different doses and exposure modes have different effects on tumor growth (**Table 1**). HBO may play a dual role in tumor angiogenesis and ROS generation.

**Table 1:** Practical application of hyperbaric oxygen.

Author	Oxygen concentration	barometric pressure	HBO exposure time per day/minutes	Days	Function	Reference
T Alagoz	100%	2.4atm	90 (30 minutes HBO+10 minutes air+30 minutes HBO+10 minutes air+30 minutes HBO)	5 days in a row	Promote tumor angiogenesis	(71)
Ingrid Moen	100%	2.5bar	90	Day 1, 4, 7	Down-regulate the MAPK pathway and reduce the density of vascular	(74)
Yasuomi Kawasoe	100%	2.5atm	60	5 times a week for 5 weeks	Enhance the effect of chemotherapy	(106)
Xin Liu	100%	2.5atm	90	Day 1, 3, 5	Enhance the immune response of PD-1 antibody to tumor	(31)
Xian Wu	>97%	2.5ata	120	3 days in a row	Relieve tumor hypoxia	(22)
Pan Wang	100%	2.5atm	90	15 days in a row	Inhibit the expression of HIF1 α and HIF2 α	(66)
Xiaoxian Wang	100%	2.5ata	90	Day 1, 2, 3, 4, 7, 10	Inhibit Cancer-Associated Fibroblasts	(64)
Shao-Yuan Chen	98%	2.5ata	90	14 days in a row	Improve tumor angiogenesis	(54)
Linda Elin Birkhaug Stuhr	>97%	2bar	90	Day 1, 4, 7	Induce apoptosis of tumor cells	(75)
Yong-Gang Wang	100%	2.5atm	60	10 days in a row	Inhibit T cell maturation	(90)
Chunxia Chen	100%	2.46atm	60	Day 1	Reduce ROS in cells and lipids and inhibit iron death	(107)
Qin Hu	–	2.5ata	90	One cycle is 7 consecutive days, with a rest of 5 days. Three cycles	Promote neural function recovery through ROS/HIF-1 α/β-catenin pathway	(108)

atm, atmospheres; ata, atmospheres absolute; MAPK, Mitogen-activated protein kinase.

## Discussion

Immunotherapy has achieved great success since its debut. It has shown strong anti-tumor activity in the treatment of solid tumors such as melanoma (109), non-small cell lung cancer (110), renal cell cancer (111), and prostate cancer (112), which has changed the pattern of tumor treatment to a certain extent. However, clinical drug resistance limits its development (6). In recent years, there have been many studies on drug resistance in immunotherapy. Esther Redin found that dasatinib increased the antitumor activity of anti-PD-1 by inhibiting the transformation of Treg cells (113). Guohao Wang believes that nano units can enhance the response to PD-L1 checkpoint blocking (114). We searched for targets and therapeutic strategies for immunotherapy resistance at gene and molecular levels, which suggested the importance of the tumor microenvironment for immunotherapy. HBO therapy has a long history. Recently, the combination of HBO with radiotherapy, chemotherapy, and photodynamic therapy has shown good therapeutic effects (115). Therefore, we may also consider combining HBO with cancer treatment to explore its impact on cancer treatment.

Most cancer patients will have an imbalance of immune system function. Considering the influence of HBO on the immune system and its potential therapeutic effect in autoimmune diseases, the combination of HBO and immunotherapy is a promising therapeutic strategy. HBO improves tumor hypoxia by down-regulating HIF1α (64). Targeting HIF1α in immunotherapy is a relatively new concept and its rationale has been well-documented by others (18, 19, 62). HIF1α is usually inactivated in normal tissues, but it is usually stable in tumor cells, regardless of oxygen tension. Targeting HIF1α has been shown to isolate immunotherapeutic effects and reduce the incidence of immune-related adverse events in preclinical models (116). HBO normalizes the vascular composition around the tumor. HBO depletes ECM collagen fibrils, collagen I, and fibronectin (63). HBO can regulate the tumor microenvironment by increasing the proportion of MI and M2 phenotype macrophages and effector memory T cells. Finally, HBO has also been found to promote the infiltration of PD-1 antibodies and T cells into solid tumors (31). But HBO therapy has not been shown clinically to affect cancer in any significant way by itself, which strongly suggests that it must be used in combination with immunotherapy. In addition, HBO enhances the therapeutic effect of antibodies in non-cancer diseases. Antibody therapy is a type of immunotherapy. Therefore, We can consider combining HBO with immunotherapy for cancer.

But HBO can also produce ROS while regulating the tumor microenvironment. Different levels of ROS in cancer treatment are a double-edged sword. The amount of ROS produced *in vivo* by HBO therapy lacks specific metrics to determine. Therefore, The suppressive effect of HBO therapy on immunotherapy also needs to be considered.

Many studies have been conducted today to overcome tumor hypoxia, such as using HBO therapy, oxygen delivery by nanocarriers (117–119), normobaric hyperoxia (62, 120), vascular normalization to enhance blood perfusion and oxygenation (121), and reduction of cellular oxygen consumption (122). These approaches have been shown to activate CTL and enhance ICBs through antibody-mediated immunotherapy. However, most of these studies exist in preclinical models and there is still a long way to go before they can be truly applied in clinical practice. For example, Normobaric hyperoxia, a relatively well-established clinical oxygenation strategy, has been found to enhance anti-tumor activity by suppressing tumor-reactive immune cells. However, HBO is not normobaric hyperoxia. HBO increases the air pressure at the same time as increasing the oxygen concentration. Stress genes are very important, and HBO can inhibit pro-inflammatory genes and affect immune response. Therefore, HBO plays an immunomodulatory role through hyperoxia and high pressure (62).

While HBO therapy is expected to overcome hypoxia by increasing the oxygen supply to the tumor tissue, its beneficial effects are varied. HBO therapy varies depending on the type of tumor, the size of the lesion, and the clinical status of the patient. Therefore, the application time, duration, and dose of HBO are very important (49). We found that in the practical application of HBO, the commonly used dose is 100% oxygen and 2.5 atm. HBO treatment for 90 minutes every day for 3-7 days may inhibit tumor growth and promote chemotherapy and immunotherapy of cancer. However, the best dose and exposure mode of HBO to promote cancer immunotherapy need further study and verification.

Malignant tumor has been considered a contraindication of HBO therapy in the past, so the application of HBO in cancer is relatively rare. Today, most studies combine HBO with radiotherapy, photodynamic therapy (11), and nano-drugs (12, 63). The combination of HBO and immunotherapy is relatively rare. We found that HBO can resist the drug resistance of immune checkpoints to a certain extent and promote the immunotherapy of cancer. This paper summarizes how HBO therapy affects cancer immunotherapy by regulating the tumor microenvironment, which provides a breakthrough point for immunotherapy and may enlighten the future direction of immunotherapy.

## Author contributions

PW, X-YW, and C-FM collected the related paper and finished the manuscript and figures. YF and D-DG gave constructive guidance and made critical revisions. All authors contributed to the article and approved the submitted version.

## Glossary

**Table:**

ICBs	Immune checkpoint blockers
CTLA-4	Cytotoxic T lymphocyte-associated antigen 4
PD-1	Programmed cell death protein 1
PD-L1	Programmed cell death ligand 1
HBO	Hyperbaric oxygen
ECM	Extracellular matrix
HIF1α	Hypoxia-inducible factor 1α
MDSC	Myeloid-derived suppressor cells
VEGF	Vascular endothelial growth factor
PDGF	Platelet-derived growth factor
CTGF	Connective tissue growth factor
CTL	Cytotoxic T lymphocyte
EMT	Epithelial-mesenchymal transition
HCC	Hepatocellular carcinoma cell
LncRNA	Long non-coding RNA
Treg	Regulatory T cells
NK	Natural killer
CAFs	Cancer-associated fibroblasts
CLL	Chronic lymphocytic leukemia
ROS	Reactive oxygen species
M1	Macrophages1
M2	Macrophages2
EGFR	Epidermal Growth Factor Receptor
PI3K	Phosphatidylinositol 3-kinase
NF-κB	Nuclear factor kappa-B
IL	Interleukin
MAPK	Mitogen-activated protein kinase
DC	Dendritic cells
TCR	T cell receptor
MHC	Major histocompatibility complex
CD	Cluster of differentiation
VHL	Von Hippel Lindau
atm	atmospheres
ata	atmospheres absolute
